# Antimicrobial-Resistant *Neisseria gonorrhoeae* of Public Health Concern, New South Wales, Australia, 2022–2024

**DOI:** 10.3201/eid3206.251399

**Published:** 2026-06

**Authors:** Liz J. Walker, Sebastiaan J. Van Hal, Cecilia Li, Steven J. Nigro, Ellen Donnan, Nathan Ryder, Monica Lahra, Janaki Amin

**Affiliations:** NSW Health, St. Leonards, New South Wales, Australia (L.J. Walker, C. Li, S.J. Nigro, E. Donnan, N. Ryder, J. Amin); Royal Prince Alfred Hospital, Sydney, New South Wales, Australia (S.J. van Hal); The University of Sydney Central Clinical School, Sydney (S.J. van Hal); The University of Sydney Institute for Infectious Diseases, Sydney (E. Donnan); Prince of Wales Hospital and Community Health Services, Randwick, New South Wales, Australia (M.M. Lahra); University of New South Wales, Sydney (M.M. Lahra); Macquarie University, Sydney (J. Amin)

**Keywords:** gonorrhea, *Neisseria gonorrhoeae*, bacteria, antimicrobial resistance, sexually transmitted infections, ceftriaxone, azithromycin, molecular epidemiology, epidemiologic monitoring, Australia

## Abstract

Antimicrobial-resistant *Neisseria gonorrhoeae* poses a serious public health threat. In Australia, *N. gonorrhoeae* isolates with ceftriaxone MICs >0.125 mg/L or azithromycin MICs >256 mg/L required follow-up by public health officials. Odds of culture-positive notifications meeting those criteria increased from 2017–2019 (n = 11) to 2022–2024 (n = 94) (odds ratio 8.58 [95% CI 4.81–17.0]). Local acquisition was frequent (78.7%). Isolates with decreased ceftriaxone susceptibility were more common in female and heterosexual patients than were isolates with high-level azithromycin resistance. We identified 9 genomically linked clusters (<15 single-nucleotide polymorphisms), 3 with sizable clonal expansion. Initial test of cure was negative for 81/94 (86.2%) 2022–2024 cases; of the rest, 9 cases had no follow-up visits, 2 were reinfected, and 2 failed initial treatment. Improving follow-up and reporting of treatment failure would strengthen case management protocols. Culture-based diagnostics remain essential to detect antimicrobial resistance, inform surveillance, and curb the rising resistance trend.

Escalating antimicrobial resistance (AMR) in *Neisseria gonorrhoeae* poses a serious public health threat, undermining treatment and disease control strategies globally. In Australia, national sexually transmitted infection (STI) management guidelines recommend collection of gonococcal culture samples from all infected sites before antimicrobial treatment is initiated (*1*). The current recommended antimicrobial treatment for uncomplicated urogenital and anorectal gonorrhea is dual therapy, comprising ceftriaxone (500 mg intramuscular injection) and oral azithromycin (1 g; 2 g if oropharyngeal infection) (*1*).

New South Wales (NSW), the most populous state in Australia, includes the city of Sydney with its ≈8 million residents. NSW has a robust passive gonococcal surveillance program that routinely performs antimicrobial susceptibility testing (AST) on all cultured gonococcal isolates collected across broad clinical settings, including sexual health clinics, general practices, and hospitals. Cultured isolates that have undergone AST represent ≈27.0% of all gonorrhea notifications in the state and were obtained from a diverse range of persons. In other surveillance programs, collection of gonococcal isolates can be limited by use of quotas, sentinel sites, and designated collection periods each year (*2*–*6*).

NSW’s comprehensive surveillance program makes it a key contributor to national monitoring. In 2024, the national proportion of gonococcal isolates with decreased susceptibility (DS) (MIC >0.125 mg/L) to ceftriaxone was 0.5% (55/10,702), representing a substantial increase from 2023 (0.2% 22/10,105); of the 2024 isolates, 40% (22/55) were reported from NSW (*7*). Similarly, gonococci with azithromycin high-level resistance (HLR) have shown increasing numbers: 9 notifications in 2022, 27 in 2023, and 46 in 2024; most (26/46 [57%]) of those isolates were from NSW (*7*). 

The increasing reports of gonococci resistance, particularly in NSW, underscore the need for enhanced knowledge in local epidemiology and transmission networks. We conducted a retrospective observational study integrating AMR data, case investigations, and genomic analyses to provide a comprehensive picture of antimicrobial-resistant gonorrhea of public health concern in NSW. Drawing on intelligence about introduced and locally circulating strains across population groups, this study aimed to provide evidence to support updates to gonorrhea treatment guidelines and strengthen public health response procedures.

## Methods

### Gonococcal Surveillance Program

In NSW, gonorrhea is a notifiable condition under the Public Health Act 2010; confirmed cases require detection of *N. gonorrhoeae* by nucleic acid amplification test (NAAT), isolation by culture, or both. All cultured isolates are referred to the World Health Organization (WHO) Collaborating Centre for Sexually Transmitted Infections and Antimicrobial Resistance at the NSW Health Pathology Laboratory (Randwick, NSW, Australia) for confirmation of identification and AST. We performed AST using Etest gradient diffusion strips (bioMérieux, https://www.biomerieux.com) to determine MIC values reported in accordance with Clinical and Laboratory Standards Institute breakpoints (*8*) *N. gonorrhoeae* isolates with ceftriaxone DS (MIC >0.125 mg/L) or azithromycin HLR (MIC >256 mg/L) or both are designated as being of public health concern (PHC) and prompt a public health response (*9*). The managing clinician is responsible for patient recall, treatment, provision of enhanced surveillance data, and contact tracing (*10*). The contact tracing lookback period is 2 months before symptom onset or before date of diagnosis for asymptomatic patients. Clinicians are advised to recall the patient for test of cure (TOC) by NAAT, collecting a swab from each site of infection (genitourinary, rectal, pharyngeal) 2 weeks after treatment completion to detect treatment failure, emerging resistance, or reinfection.

Twelve public and private laboratories support gonorrhea surveillance by providing aggregate annual gonorrhea testing data from NAAT and culture. Those laboratories are estimated to account for 88.0% of all gonorrhea testing in NSW, ensuring comprehensive monitoring of diagnostic activity across the state.

### Case Identification and Statistical Analysis

We extracted cases of antimicrobial-resistant gonorrhea of PHC notified during January 1, 2022–December 31, 2024, from the NSW Notifiable Conditions and Information Management System (NCIMS). We also extracted historic notifications for chlamydia, gonorrhea, and syphilis that shared the unique person identifier with an AMR notification. We derived sexual risk group from sex at birth and reported sexual exposures during the exposure period; we classified cases in men who reported sex with men only as men who have sex with men (MSM), cases in men who reported sex with both sexes as bisexual, and cases in men and women who reported sex only with the opposite sex as heterosexual. We categorized country of acquisition of infection in accordance with WHO region (*11*). To examine changes in notification patterns over time, we compared data for 2017–2019 (pre–COVID-19 pandemic) and 2022–2024 (post–COVID-19 pandemic). We compared the characteristics of notifications identified as ceftriaxone DS with notifications of azithromycin HLR. We used Fisher exact and Wilcoxon rank-sum test to test significance of comparisons; we chose a 2-sided α of p<0.05 as the threshold for statistical significance. We analyzed epidemiologic data using R version 4.4.1 (The R Project for Statistical Computing, https://www.r-project.org), ggplot version 3.5.1 (https://github.com/tidyverse/ggplot2), and gtsummary version 2.0.2 (https://github.com/ddsjoberg/gtsummary).

### Whole-Genome Sequencing

We sent all isolates of PHC for whole-genome sequencing (WGS) using an Illumina platform (https://www.illumina.com) at NSW Health Pathology (Camperdown, NSW, Australia). We used the constructed assemblies to define AMR genes and multilocus sequence types (ST) (*12*). We excluded 1 isolate that did not meet quality metrics. We conducted a network analysis of the mapped single nucleotide matrix against reference isolate FA1090 (GenBank accession no. NC_002964.2) using the R packages tidygraph version 1.3.1 (https://github.com/thomasp85/tidygraph) and ggraph version 2.2.1 (https://github.com/thomasp85/ggraph) to explore associations between isolates using a single-nucleotide polymorphism maximum threshold of 15.

Ethics approval for this investigation was not required because it was undertaken for purposes under the provisions of the Public Health Act 2010 as part of routine public health surveillance and reporting under the governance of Health Protection NSW. No additional data were collected for the purpose of this report, and no personal identifiable data were included. We managed data in accordance with the National Health and Medical Research Council’s Ethical Considerations in Quality Assurance and Evaluation Activities (*13*).

## Results

During 2022–2024, gonorrhea notifications increased 36.5%, from 10,288 to 14,045, whereas gonorrhea testing increased 13.1% from 775,161 to 876,447 (Table 1). The percentage of gonorrhea notifications accompanied by positive culture remained relatively stable at 27.0%, with a slight predominance in male (28.1%) versus female (22.6%) patients. Of the 9,941 culture-positive notifications, 94 (0.9%) met the definition of PHC, an overall increase from 0.5% to 1.3%, although the trend was not linear (Table 1). For comparison, in the prepandemic period before the study (2017–2019), such notifications were rare; 11 were reported, 4 of ceftriaxone DS and 7 azithromycin HLR (Table 2). The rise to 1.3% cases of PHC was accompanied by a markedly higher likelihood that culture-positive gonorrhea notifications in 2022–2024 met the definition for an AMR notification of PHC, compared with those from 2017–2019 (odds ratio 8.58, 95% CI 4.81–17.0; p<0.001). No cases of combined resistance (ceftriaxone DS and azithromycin HLR) were notified in the study period.

**Table 1:** Tests and notifications of *Neisseria gonorrhoeae*, New South Wales, Australia, 2022–2024*

Characteristic	No. (%)			
	2022	2023	2024	Total
All positive and negative test results†	775,161 (100)	868,741 (112.1)‡	876,447 (113.1)‡	2,520,349 (NA)
All *N. gonorrhoeae* notifications§	10,288 (100.0)	12,453 (100.0)	14,045 (100.0)	36,786 (100.0)
F	2,066 (20.1)	2,651 (21.3)	2,789 (19.9)	7,506 (20.4)
M	8,194 (79.6)	9,759 (78.4)	11,216 (79.9)	29,169 (79.3)
*N. gonorrhoeae* notifications with positive culture§¶	2,734 (26.6)	3,558 (28.6)	3,649 (26.0)	9,941 (27.0)
F	455 (22.0)	631 (23.8)	609 (21.8)	1,695 (22.6)
M	2,267 (27.7)	2,914 (29.9)	3,028 (27.0)	8,209 (28.1)
*N. gonorrhoeae* AMR notifications of PHC#	29 (1.1)	17 (0.5)	48 (1.3)	94 (0.9)
F	6 (1.3)	3 (0.5)	9 (1.5)	18 (1.1)
M	23 (1.0)	14 (0.5)	39 (1.3)	76 (0.9)

*AMR, antimicrobial resistance; NA, not applicable; PHC, public health concern. †Aggregate gonorrhea test results by nucleic acid amplification test and culture from 12 laboratories. Those laboratories account for an estimated 88.0% of all gonorrhea testing in New South Wales. ‡Percentage calculated from baseline tests in 2022. §Includes persons whose sex was not reported. ¶Percentage calculated from all *N. gonorrhoeae* notifications. #Percentage calculated from *N. gonorrhoeae* culture notifications.

**Table 2:** Tests and notifications of *Neisseria gonorrhoeae*, New South Wales, Australia, 2017–2019*

Characteristic	No. (%)			
	2017	2018	2019	Total
All positive and negative test results†	903,272 (100)	934,944 (103.5)‡	969,429 (107.3)‡	2,807,645 (NA)
All *N. gonorrhoeae* notifications§	9,192 (100.0)	10,529 (100.0)	11,671 (100.0)	31,392 (100.0)
F	1,512 (16.4)	1,904 (18.10)	2,397 (20.5)	5,813 (18.5)
M	7,644 (83.2)	8,588 (81.60)	9,232 (79.1)	25,464 (81.1)
*N. gonorrhoeae* notifications with positive culture§¶	2,812 (30.6)	3,305 (33.3)	3,583 (30.7)	9,700 (31.5)
F	368 (24.3)	515 (27.0)	544 (22.7)	1,427 (24.5)
M	2,427 (31.8)	2,979 (34.7)	3,027 (32.8)	8,433 (33.1)
*N. gonorrhoeae* AMR notifications of PHC#	3 (0.1)	4 (0.1)	4 (0.1)	11 (0.1)
F	0	1 (0.2)	0	1 (0.1)
M	3 (0.1)	3 (0.1)	4 (0.1)	10 (0.1)

*AMR, antimicrobial resistance; NA, not applicable; PHC, public health concern. †Aggregate gonorrhea test results by nucleic acid amplification test and culture from 12 laboratories. Those laboratories account for an estimated 88.0% of all gonorrhea testing in New South Wales. ‡Percentage calculated from baseline *N. gonorrhoeae* tests in 2017. §Includes persons whose sex was not reported. ¶Percentage calculated from all *N. gonorrhoeae* notifications. #Percentage calculated from *N. gonorrhoeae* culture notifications.

### Case Characteristics

**Figure 1:**
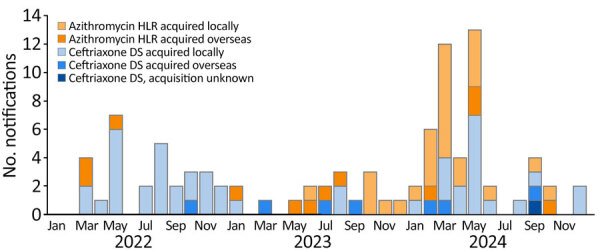
Epidemiologic curve of notifications of antimicrobial-resistant *Neisseria gonorrhoeae* of public health concern, New South Wales, Australia, 2022–2024. Shading indicates type of resistance and acquisition source. DS, decreased susceptibility; HLR, high-level resistance.

Most (74/94 [78.7%] cases) of the notifications of antimicrobial-resistant gonorrhea of PHC were acquired in Australia (Figure 1). Of those, 40 were notified in 2024. The remaining cases were acquired overseas from countries in the Western Pacific (n = 9), the Americas (n = 6), Europe (n = 3), and Africa (n = 1) WHO regions.

### Case Comparison by AMR Profile

We detected notable differences between the ceftriaxone DS and azithromycin HLR groups (Table 3). The ceftriaxone DS group had a greater percentage of female patients (31.5% vs. 2.5%; p<0.001) and persons who reported heterosexual exposures (72.2% vs. 5.0%, p<0.001), compared with the azithromycin HLR group. Most cases acquired their infection in Australia (85.2%); however, overseas acquisition, when reported, occurred exclusively in the Western Pacific region (13.0%). Cases were evenly distributed between sexual health clinics (51.9%) and general practice (40.7%); the remaining 7.4% were diagnosed in hospitals.

**Table 3:** Characteristics of antimicrobial-resistant *Neisseria gonorrhoeae* cases of public health concern, New South Wales, Australia, 2022–2024*

Characteristic	Overall, N = 94	Ceftriaxone DS, n = 54	Azithromycin HLR, n = 40	p value
Patient age at diagnosis, median (IQR)	33 (25–42)	36 (24–43)	30 (27–36)	0.12†
Sex at birth				**<0.001**‡
F	18 (19.1)	17 (31.5)	1 (2.5)	
M	76 (80.9)	37 (68.5)	39 (97.5)	
Sexual risk group				**<0.001**‡
Heterosexual	41 (43.6)	39 (72.2)	2 (5.0)	
MSM	49 (52.1)	14 (25.9)	35 (87.5)	
Bisexual	4 (4.3)	1 (1.9)	3 (7.5)	
Previous STI				**<0.002**‡
Y	47 (50.0)	19 (35.2)	28 (70.0)	
N	47 (50.0)	35 (64.8)	12 (30.0)	
Most recent previous STI occurrence§				0.7‡
Co-infection	18 (38.3)	7 (36.8)	11 (39.3)	
<1 y	22 (46.8)	8 (42.1)	14 (50.0)	
>1 y	7 (14.9)	4 (21.1)	3 (10.7)	
None	47	35	12	
No. previous STIs, median (IQR)	3 (1–5)	2 (1–4)	4 (2–6)	0.11‡
Unknown	47	35	12	
Sex worker or client or SOPV attendance				0.13‡
Yes	15 (16.0)	10 (18.5)	5 (12.5)	
No	38 (40.4)	17 (31.5)	24 (60.0)	
Unknown	41 (43.6)	27 (50.0)	14 (35.0)	
Signs and symptoms				0.095‡
Symptomatic	63 (67.0)	38 (70.4)	25 (62.5)	
Asymptomatic	24 (25.5)	10 (18.5)	14 (35.0)	
Unknown	7 (7.4)	6 (11.1)	1 (2.5)	
Site of infection				**<0.001**‡
Genitourinary tract	58 (61.7)	44 (81.5)	14 (35.0)	
Rectal	18 (19.1)	3 (5.6)	15 (37.5)	
Pharynx	12 (12.8)	4 (7.4)	8 (20.0)	
Eye	1 (1.1)	1 (1.9)	0	
Multiple sites	5 (5.3)	2 (3.7)	3 (7.5)	
Source of infection				**<0.001**‡
Australia	74 (78.7)	46 (85.2)	28 (70.0)	
Africa	1 (1.1)	0	1 (2.5)	
Americas	6 (6.4)	0	6 (15.0)	
Europe	3 (3.2)	0	3 (7.5)	
Western Pacific	9 (9.6)	7 (13.0)	2 (5.0)	
Unknown	1 (1.1)	1 (1.9)	0	
Partners within 2-month lookback				
Total no. partners, median (IQR)	2 (1–5)	1 (1–2)	5 (2–9)	**<0.001**‡
Unknown	26	17	9	
Diagnosing facility				**0.001**‡
General practice	28 (29.8)	22 (40.7)	6 (15.0)	
Sexual health clinic	62 (66.0)	28 (51.9)	34 (85.0)	
Hospital	4 (4.3)	4 (7.4)	0	
Locality				**0.030**‡
Metropolitan	90 (95.7)	54 (100.0)	36 (90.0)	
Regional	4 (4.3)	0	4 (10.0)	
Outcome of initial test of cure				0.14‡
Negative	81 (86.2)	44 (81.5)	37 (92.5)	
Positive	4 (4.3)	2 (3.7)	2 (5.0)	
No follow-up visits	9 (9.6)	8 (14.8)	1 (2.5)	

*Values are no. (%) except as indicated. Ceftriaxone DS is defined as MIC >0.125 mg/L; azithromycin HLR is defined as MIC >256 mg/L. Bold text indicates statistical significance. DS, decreased susceptibility; HLR, high-level resistance; IQR, interquartile range; MSM, men who have sex with men; SOPV, sex on premises venue; STI, sexually transmitted infection. †By Wilcoxon rank-sum test. ‡By Fisher exact test. §Categorizes the most recent STI occurrence.

In contrast, case-patients with azithromycin HLR were predominantly male (97.5% vs. 68.5% cases with ceftriaxone DS; p<0.001) and reported sexual exposures with men only (MSM) (87.5% vs. 25.9%; p<0.001). Previous STIs were more common in this group (70.0% vs. 35.2%; p<0.002), and of persons with a previous STI, the median was 4 versus 2 infections for ceftriaxone DS (p = 0.11). Infection site distribution also differed between those with azithromycin HLR and ceftriaxone DS (p<0.001). Azithromycin HLR cases were more evenly distributed across infection sites: rectal (37.5%), genitourinary tract (35.0%,) and pharynx (20.0%), whereas the genitourinary tract was most common (81.5%) in the ceftriaxone DS group, followed by pharynx (7.4%), rectal (5.6%), and eye (1.9%) specimens. Source of infection was predominantly within Australia (70.0%); however overseas acquisition also occurred across multiple WHO regions: Europe (n = 6), the Americas (n = 3), Africa (n = 1) and the Western Pacific (n = 1). Where known, the median number of partners across the lookback period was 5 for the azithromycin HLR group versus 1 for the ceftriaxone DS group (p<0.001). Sexual-health clinics diagnosed 85.0% of cases.

Outcome of initial TOC did not differ between the ceftriaxone DS and azithromycin HLR groups; most returned a NAAT-negative result >2 weeks after treatment completion (81.5% ceftriaxone DS vs. 92.5% azithromycin HLR; p = 0.14). Both groups reported a small number of patients who returned a NAAT-positive result (2/54 vs. 2/40) (Table 3). In the ceftriaxone DS group, 1 case-patient with a genitourinary infection tested NAAT positive on the initial TOC from the same site but subsequently tested NAAT negative after a repeat round of dual therapy. Both isolates were identical on genomic analysis. Another case-patient with a genitourinary infection underwent multisite screening for the initial TOC and tested NAAT positive on the pharyngeal swab specimen but cleared the infection with repeat dual therapy with increased azithromycin (2 g orally). Unfortunately, no culture nor genomics were performed. In the azithromycin HLR group, 2 patients were believed to be reinfected based on patient history.

### Genomic Analysis and Clustering of Isolates

**Figure 2:**
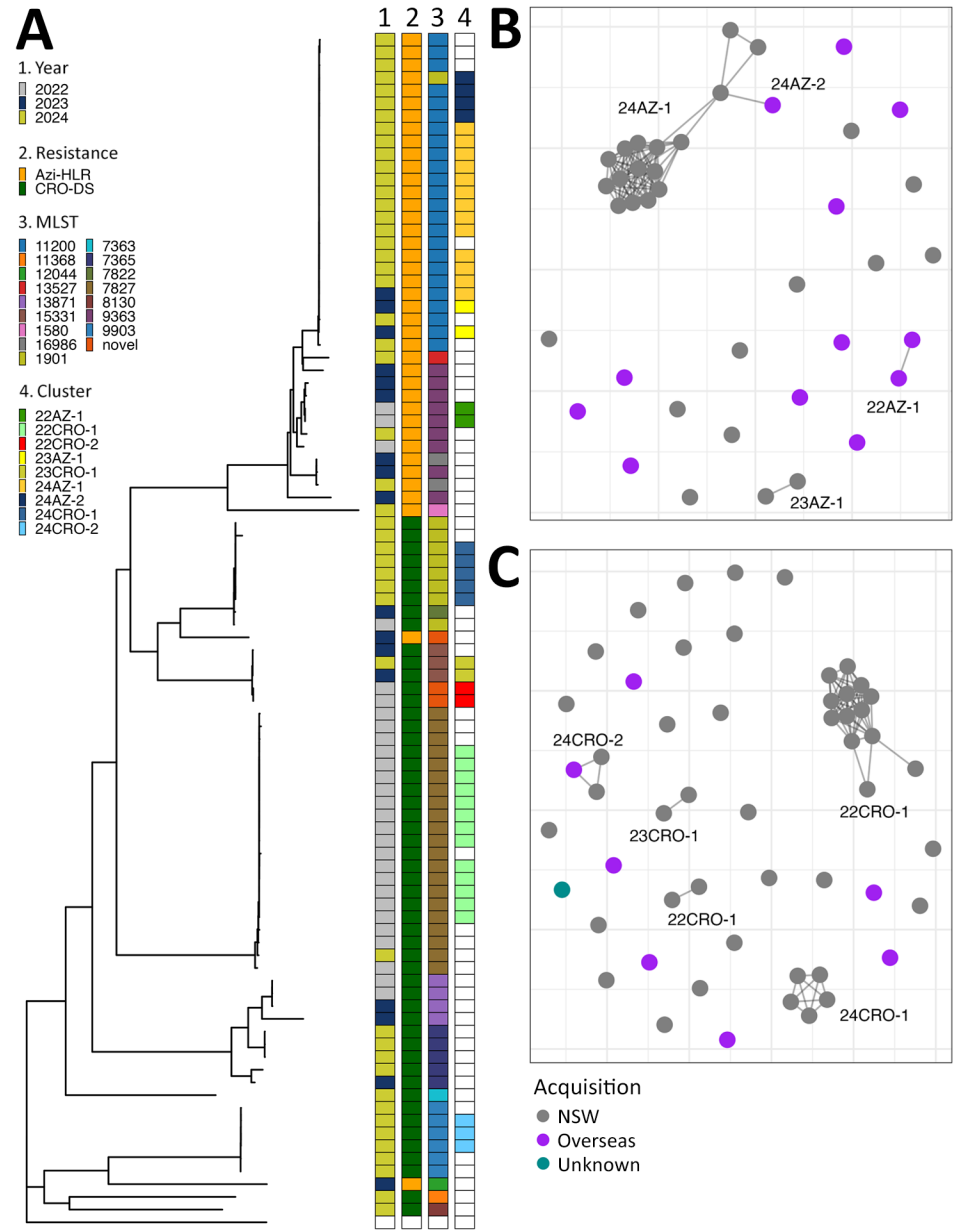
Phylogenetic tree and antimicrobial resistance network analysis from study of antimicrobial-resistant *Neisseria gonorrhoeae* of public health concern, New South Wales, Australia, 2022–2024. A) Phylogenetic tree and isolate data. Ninety-three of the 94 isolates met the quality mapping criteria. B) Network analysis of cases with high-level resistance to azithromycin, with nodes colored according to acquisition source. C) Network analysis of cases with decreased susceptibility to ceftriaxone, with nodes colored according to acquisition source. Azi, azithromycin; CRO, ceftriaxone; DS, decreased susceptibility; HLR, high-level resistance; MLST, multilocus sequence type.

Of the 94 isolates, 93 met the quality mapping criteria. Azithromycin HLR was associated with the A2059G mutation in the 23S rRNA gene in 39/40 (97.5%) cases, whereas ceftriaxone DS was linked to the presence of the *penA*-60.001 allele in 24/53 (45.0%) cases. We noted 17 distinct multilocus ST with evidence of clonal expansion across the 3 years (Figure 2).

We identified 9 clusters on the basis of network analysis using a threshold of <15 single-nucleotide polymorphisms (Table 4; Figure 2). We identified 2 large ceftriaxone DS clusters, 1 of sequence type (ST) 7827, consisting of 13 cases (22CRO-1), all locally acquired; 5 case-patients reported recent links to commercial sex work. The cluster was predominantly (92.0%) heterosexual, with 5 female and 8 male case-patients. After that local spread, the clone was infrequently detected after 2022. Subsequent ceftriaxone DS isolates in 2023–2024 were of greater concern because 22/28 cases harbored the *penA*-60.001 allele. Two clusters were detected in 2024. The largest cluster consisted of 5 ST1901 infections in heterosexual cases that were all locally acquired; 2 case-patients reported links to sex work. In contrast, the ST9903 cluster consisted of 3 MSM, 1 of whom acquired the infection in Cambodia. Those cases were diagnosed within a small geographic area.

**Table 4:** Summary of antimicrobial-resistant *Neisseria gonorrhoeae* cluster groups, New South Wales, Australia, 2022–2024*

Cluster ID	Size	MLST	*penA *allele type	X23 rRNA	Sex at birth, no. (%)	Sexual risk exposure group, no. (%)	Place of acquisition, no. (%)	SW or client or SOPV, no. (%)	Median no. contacts (range)
22AZ-1	2	9363	2.001	A2059G	2 M (100.0)	2 MSM (100.0)	2 OS (100.0)	0	Unknown
23AZ-1	2	11200	2.001	A2059G	2 M (100.0)	2 MSM (100.0)	2 local (100.0)	0	2.5 (1–4)
24AZ-1	13	11200	2.001	A2059G	13 M (100.0)	12 MSM (92.3), 1 BI (7.7)	13 local (100.0)	2 (15.4)	3.5 (1–30)
24AZ-2	4	11200	2.001	A2059G	4 M (100.0)	3 MSM (75.0), 1 BI (25.0)	3 local (75.0), 2 OS (25.0)	1 (25.0)	9.0 (1–15)
22CRO-1	13	7827	13.001	NA	5 F (38.5), 8 M (61.5)	12 HET (92.3), 1 MSM (7.7)	13 local (100.0)	5 (38.5)	1.0 (1–5)
22CRO-2	2	15331	18.001	NA	2 M (100.0)	2 MSM (100.0)	2 local (100.0)	0	3.0†
23CRO-3	2	15331	18.001	NA	2 M (100.0)	2 MSM (100.0)	2 local (100.0)	0	6.0†
24CRO-1	5	1901	60.001	NA	2 F (40.0), 3 M (60.0)	5 HET (100.0)	5 local (100.0)	2 (40.0)	1.0 (1–2)
24CRO-2	3	9903	60.001	NA	3 M (100.0)	3 MSM (100.0)	2 local (66.6)	0	1.5 (1–2)
							1 OS (33.3		
Total	46	NA	NA	NA	7 F (15.2)	17 HET (37.0)	42 local (91.3)	10 (22.7)	2.0 (1–30)
					39 M (84.8)	27 MSM (58.7), 2 BI (4.3)	4 OS (8.7)		

*BI, bisexual; HET, heterosexual; ID, identification; local, Australia acquisition; MLST, multilocus sequence type; MSM, men who have sex with men; NA, not applicable; OS, overseas acquisition; SOPV, sex on premises venue; SW, sex worker. †Median number of contacts (based on 1 available value).

We detected 2 separate but connected ST11200 clusters, 24AZ-1 (n = 13) and 24AZ-2 (n = 4), in the azithromycin HLR notifications. All cases were locally acquired except for 1 from China. All 17 case-patients were male, 88.2% MSM and 11.7% bisexual. Two reported attendance at sex on premises venues, and 1 had links to sex work. We observed 2 additional clusters, 22AZ-1 ST9363 in 2 MSM cases, both overseas acquired in South America, and 23AZ-1 ST11200 in 2 MSM cases acquired locally.

Genomics linked 46 (49.5%) of 93 notifications. In contrast, epidemiologic links were reported for 5 (5.3%) of 94 notifications. Of note, 2 cases reported an epidemiologic link that was not confirmed by WGS. The discordance between genomic links and self-reported epidemiologic links is not surprising, given the nature of self-reported participation in commercial sex work and attendance at sex on premises venues. Apart from a significantly higher proportion of locally acquired cases within clusters (42 [91.3%] vs. 32 [66.7%] cases; p = 0.047), we determined no statistically significant differences in case characteristics between clustered and nonclustered cases (data not shown).

## Discussion

Culture-positive notifications of gonorrhea with ceftriaxone DS or azithromycin HLR were rare before the COVID-19 pandemic and increased notably postpandemic (2022–2024), highlighting a concerning upward trend. Of greater concern is that ≈80.0% of postpandemic antimicrobial-resistant gonorrhea notifications were locally acquired; clusters arose infrequently from imported cases. That finding suggests that resistant gonococcal strains are actively circulating in communities of sexually active persons in NSW; although clusters in the study were relatively small, there is a risk for future expansion. The risk is perhaps highest among heterosexual populations; the infection can remain asymptomatic in 80.0% of women (*1*), and STI testing rates are lower in heterosexual persons. Lifetime uptake of STI testing is 12.8% in cisgender heterosexual male persons and 15.4% in cisgender heterosexual females versus 38.8% in gay and bisexual and other MSM (GBMSM) and 54.4% in sex workers (*14*). This concept is supported by a previous study in Australia that demonstrated *N. gonorrhoeae* genomic clusters with a higher ratio of women to men were more likely to persist compared with clusters in GBMSM (*15*). Despite higher STI screening rates in GBMSM, the risk for gonorrhea infection and transmission remains high. Uptake of preexposure prophylaxis (PrEP) for HIV prevention among Sydney GBMSM has been substantial (34.9% in 2020 to 53.2% in 2024) (*16*); although evidence of whether PrEP leads to higher STI rates is mixed, data from the NSW GBMSM community suggests STIs were increasing before its introduction (*17*). Instead, the rise in multiple sex partners may be contributing to increased gonorrhea notifications; 26.4% of respondents reported sex with 6–20 men and 16.2% reported sex with >20 men in the 6 months before the Sydney GBQ+ Community Survey in 2024 (up from 23.4% in the 6–20 group and 12.5% in the >20 group in 2020) (*16*). The observed increase in the percentage of GBMSM reporting multiple sexual partners in the community may have contributed to the expansion of 24AZ-1 cluster, in which cases reported 1–30 contacts.

Although antimicrobial-resistant isolates of PHC in our study remain uncommon (<1.0% of culture-positive *N. gonorrhoeae* notifications), that is likely to change with factors such as increased use of doxycycline postexposure prophylaxis (doxy-PEP). In March 2024, the Australia consensus statement on doxy-PEP to prevent syphilis, chlamydia, and gonorrhea among GBMSM was published, after a collaborative process involving community representatives and experts in clinical care, research, and public health (*18*). The statement acknowledged that doxy-PEP was less likely to be effective for gonorrhea prevention because of high rates of tetracycline resistance in Australia gonococcal isolates (2022–2024, 31.0%–45.0%; in NSW, 20.0%–31.5%) (*7*,*19**,**20*). By 2025, data from the Sydney GBQ+ Community Survey reported 12.3% of respondents had used doxy-PEP <6 months before the survey for the prevention of bacterial STIs, predominantly syphilis (*21*). Genomic analysis of antimicrobial-resistant gonococcal isolates indicates that mutations associated with tetracycline resistance are often co-selected alongside mutations known to cause resistance to other antimicrobial drugs, which are spread throughout successful clonal lineages (*22*). Therefore, strengthening AMR surveillance and TOC practices and documenting treatment failures are essential.

Although return for TOC was high in our study (86.2%), we found it concerning that 2 treatment failures were identified (both ceftriaxone DS cases: 1 genitourinary and 1 pharyngeal). Standard operating procedures for management of treatment failures are essential to ensure consistent reporting of second-round prescribing practices and appropriate referral of those culture-positive isolates for WGS. Where warranted, complex cases could be escalated to a clinical expert group for specialized advice. Approaches to minimize patients who do not complete follow-up visits could include providing pathology requests, especially in general practice settings at the time of treatment; offering self-collection swab kits; distributing patient information sheets; and ensuring services are provided at no cost to the patient. More broadly, we recommend that national treatment guidelines are regularly reviewed in conjunction with local epidemiology and WGS data.

AMR patterns in gonorrhea observed in NSW reflect global trends. The Western Pacific region is a recognized reservoir of gonococcal resistance; Cambodia reported ceftriaxone DS rates of 31%–38%, Vietnam 27%–28%, and China 8%–9%, including ≈25% in some provinces (*23*–*28*). Patient risk factors contributing to that resistance pattern are sparsely reported from these countries; however, a UK study found all of their local ceftriaxone DS cases were in heterosexuals and ≈90% of overseas acquired cases were associated with travel to the Western Pacific region or adjacent countries (*29*). In contrast, the epidemiology and geographic distribution of azithromycin HLR seems less well defined, with multiple sustained transmission events reported across the Americas and Europe (*30*–*33*).

A limitation of our study was the lack of data on contact tracing outcomes and subsequent transmission. In NSW, contact tracing is the responsibility of the managing clinician, and most clinicians support patients to self-inform their contacts. Provider-initiated contact tracing can be an alternative; however, potential drawbacks are privacy and confidentiality concerns and the potential to damage the patient-provider relationship. Of interest, a public health–led gonorrhea contact tracing intervention in Canada found that the provider-initiated method identified a significantly higher number of female than male cases during the intervention period (*34*). Unfortunately, in our study, many cases reported anonymous contacts, making both methods ineffective to effectively curb transmission. Efforts would be better focused on promoting diagnosis by culture, STI screening, improving sexual health education and awareness of antimicrobial resistance, and ensuring completion of TOC for all antimicrobial-resistant gonorrhea notifications of PHC.

Last, although national guidelines recommend collecting culture samples from all infected sites, not all notifications in NSW had a culture result. Culture success rates are greater for symptomatic infections, which have a higher bacterial load, than for asymptomatic infections, and vary by site of infection (*35*). In addition, factors such as bacterial viability, swab technique, transport medium, and transport time all influence culture success. Although we did not have data on the number of cultures ordered in NSW, we do have data on the *N. gonorrhoeae* culture-positive rate. The culture rate from general practice is lower than from sexual health clinics (19.1% vs. 44.6%) (unpublished data from operational audit). Collectively, those findings suggest that while culture collection practices can be improved, achieving culture positivity for all notifications is not feasible.

In summary, notifications of antimicrobial-resistant gonorrhea of PHC are rapidly rising in Australia and elsewhere. Where possible, culture-based surveillance with robust case management protocols remains the best approach to detect and minimize transmission of resistant gonococcal infection.
